# Psychometric Properties of the German Version of the Pulmonary-Specific Quality-of-Life Scale in Lung Transplant Patients

**DOI:** 10.3389/fpsyt.2019.00374

**Published:** 2019-05-31

**Authors:** Mariel Nöhre, Özgür Albayrak, Jan Brederecke, Laurence Claes, Dirk Smits, Igor Tudorache, Martina de Zwaan

**Affiliations:** ^1^Department of Psychosomatic Medicine and Psychotherapy, Hannover Medical School, Hannover, Germany; ^2^Hannover Medical School, Biomedical Research in Endstage and Obstructive Lung Disease Hannover (BREATH), German Center for Lung Research (DZL), Hannover, Germany; ^3^Faculty of Psychology and Educational Sciences, KU Leuven, Leuven, Belgium; ^4^Faculty of Medicine and Health Sciences, University of Antwerp, Antwerp, Belgium; ^5^Research Department, Odisee University College, Brussels, Belgium; ^6^Department of Cardiac, Thoracic, Transplant, and Vascular Surgery, Hannover Medical School, Hannover, Germany

**Keywords:** lung transplantation, quality of life, health-related quality of life, pulmonary-specific quality-of-life scale, pulmonary disease

## Abstract

The Pulmonary-Specific Quality-of-Life Scale (PQLS) is a validated self-report questionnaire assessing health-related quality of life (HRQoL) in patients with end-stage lung disease awaiting lung transplantation. The aim of our study was to evaluate the psychometric properties of the German version of the PQLS. One hundred and forty patients awaiting lung transplantation (55% men) with a median age of 53 years [Interquartile range (IQR) 13] answered the PQLS. A group of the participants (*n* = 43) was evaluated again 1 year later after transplantation. A confirmatory factor analysis (CFA) of the PQLS was conducted to test the three-factor structure of the PQLS. We examined the internal consistency of the scales using Cronbach’s *α*. Convergent validity was explored through correlations with generic measures of HRQoL [Short-Form 8 Health Survey (SF-8), 10-item quality of life (QoL) scale], measures of depression (nine-item Patient Health Questionnaire-Depression Scale), anxiety (Generalized Anxiety Scale), and measures of lung disease severity (supplemental oxygen use, stairway steps). In the group of 43 patients assessed before and after transplantation, sensitivity to change was explored. The CFA confirmed the three-factor model with an acceptable fit. The PQLS total and the three subscale scores “task interference,” “psychological,” and “physical” showed acceptable internal consistency. The PQLS and its subscales showed a significant negative correlation with the 10-item QoL measure and the physical component score of the SF-8, whereas the mental component score of the SF-8 showed a significant negative correlation only with the PQLS subscale “psychological.” Negative correlation was found due to the opposed alignment of the PQLS compared to the 10-item QoL and the SF-8. Symptoms of depression and anxiety were significantly and positively correlated with the subscale “psychological.” Measures of lung disease severity also exhibited a significant positive correlation with the subscales “task interference” and “physical” but not “psychological.” In patients 1 year after a successful transplantation, the PQLS scores were significantly reduced by 50%. The three-factor structure of the PQLS could be replicated using CFA. The results indicate good reliability, validity, and sensitivity to change of the German version of the PQLS.

## Introduction

For the evaluation of treatment effectiveness in lung transplantation, quality of life (QoL) has become a meaningful clinical endpoint (1, 2). It is recommended that success in lung transplantation should also be defined by patient-centered outcomes such as health-related quality of life (HRQoL) in addition to survival and transplant rates (1). While survival and transplant rates are objective parameters, they give no information regarding the patient’s well-being. It is well known that HRQoL is an important psychosocial parameter which might differ from medical parameters and the clinician’s perspective (1). There are different ways to assess HRQoL. Information on health status in patients before and after lung transplantation is often obtained using generic measures of HRQoL, e.g., The Short Form 36 health survey (SF-36) (3) and Euroqol (4). Even though generic measures allow comparison with other diseases, they are not specific to patients with advanced lung disease. For example, these patients often experience significant physical impairments, e.g., dyspnea, and are frequently dependent on high-volume supplemental oxygen (1). Generic HRQoL scales may not be sensitive enough to capture and quantify these unique effects of lung diseases on QoL (2, 5). In addition, patients’ QoL after successful transplantation may be limited by other than disease-specific factors which might lead to improvements in disease-specific but not in generic HRQoL measures. Also, disease-specific instruments might be more sensitive to change and might be more sensitive to small differences after or between treatments. From a clinical perspective, looking at the individual items of a disease-specific measure could be helpful to get a better understanding of the patients’ specific struggles in everyday life. This information might be helpful to offer specialized support.

Whereas there are lung-specific instruments available to assess HRQoL in patients with mild forms of lung diseases (6, 7) and with specific pulmonary diseases such as asthma and chronic obstructive pulmonary disease (8, 9), only few instruments are available assessing pulmonary HRQoL on a multidimensional level in patients with end-stage lung disease awaiting lung transplantation. The Pulmonary-Specific Quality-of-Life Scale (PQLS) was specifically developed by Napolitano et al. (10) to evaluate lung-specific aspects of QoL in patients awaiting lung transplantation. The psychometric properties of the original English version have been evaluated by Hoffman et al. (2). Through exploratory factor analysis, three PQLS subscales (“physical,” “psychological,” and “task interference”) could be identified.

To the best of our knowledge, no authorized validated German version of the PQLS is available so far. Hence, the aim of the present study was to examine the psychometric properties of the German version of the PQLS. We conducted a confirmatory factor analysis (CFA) of the PQLS to test the three-factor structure. We examined the internal consistency of the scales by means of Cronbach’s α. We then explored convergent validity with regard to generic measures of HRQoL, measures of depression and anxiety, and measures of lung disease severity. Finally, in a subgroup, we investigated the sensitivity to change of the PQLS 1 year after lung transplantation.

## Methods

### Participants

Between January 2016 and December 2017, 150 patients presenting for psychosocial evaluation prior to enlistment for lung transplantation were included in the study. As part of their assessment, they filled out several questionnaires. The participants had to be at least 18 years of age and have sufficient German language skills in order to understand the content of the questions. For a group of participants (*n* = 43), a 1 year follow-up after transplantation was already available. The study was approved by the Institutional Ethics Board of Hannover Medical School (no 3120-2016), and all patients gave written informed consent.

### Assessment Instruments

#### Pulmonary-Specific Quality-of-Life Scale

The Pulmonary-Specific Quality-of-Life Scale (PQLS) is a self-report questionnaire assessing QoL in patients with end-stage lung diseases awaiting lung transplantation (2, 10). The scale consists of 25 items which are rated on a five-point-Likert-scale ranging from 1 (“not at all”) to 5 (“most of the time”). A total score between 25 and 125 can be reached with higher values indicating lower HRQoL. Three subscales (“task interference,” “psychological,” and “physical”) were identified in the original English version of the PQLS (2). The subscale “task interference” (eight items, e.g., “Because of my lung disease, I have had to limit many of the activities that I enjoy”) focuses on occupational and social functioning, the subscale “psychological” (seven items, e.g., “I have been feeling down, blue, or depressed”) assesses mental and psychological aspects, and the subscale “physical” (four items, e.g. “I am able to walk up a flight of stairs without getting winded”) evaluates physical functioning. Six items did not load on any factors; thus, the total scale is also reported as suggested by the original authors of the scale. In our study, a German translation of the questionnaire was used, which has been created with the approval of the original authors of the PQLS. The German translation and back-translation of the original English version were performed by a licensed translator (Translaw, Oxford, GB), who also checked the backwards translation for discrepancies against the original version. The German version is available as a supplementary file; the English original is published in Hoffman et al. (2).

#### Generic Health-Related Quality of Life

To measure generic HRQoL, the Short-Form 8 Health Survey (SF-8), a short version of the SF-36 Health Survey, was used (11–13). The two summary scales, i.e., the Physical Component Scale (PCS) and the Mental Component Scale (MCS), were used. Both scales are standardized combined scores with a mean of 50 and a standard deviation of 10 in the US general population. Cronbach’s α for the total SF-8 in the present study sample was 0.73.

Additionally, participants were asked to rate their general satisfaction with their current QoL on a scale ranging from 0 to 10, with 0 meaning totally unsatisfied and 10 meaning “perfectly satisfied.”

#### Symptoms of Depression and Anxiety

To assess symptoms of depression, the German version of the nine-item Patient Health Questionnaire-Depression Scale (PHQ-9) (14, 15) was used. The scale consists of nine items. Each item is scored on a four-point Likert-scale ranging from 0 (“not at all”) to 3 (“nearly every day”). A total score between 0 and 27 can be reached. Higher values indicate a higher level of depressive symptoms. A total score ≥10 has been established as clinically relevant for screening purposes. Cronbach’s α was 0.76.

The German version of the Generalized Anxiety Scale (GAD-7) was used to evaluate symptoms of anxiety (16, 17). Each of the seven items is scored on a four-point Likert scale ranging from 0 (“not at all”) to 3 (“nearly every day”), yielding a total score between 0 and 21. Higher total scores correspond with higher levels of anxiety symptoms. Values above a cutoff ≥10 indicate a clinically relevant level of anxiety. Cronbach’s α in the present sample was 0.78.

#### Demographics and Measures of Disease Severity

Patients were asked to report their age, sex, years of completed education, and partnership status. Depending on the lung disease, four groups were created: obstructive lung disease (e.g., chronic obstructive lung disease/emphysema, bronchiectasis), restrictive lung disease (e.g., pulmonary fibrosis), cystic fibrosis, and other lung diseases (e.g., pulmonary vascular disease, idiopathic pulmonary arterial hypertension, and sarcoidosis). Functional capacity was assessed with supplemental oxygen use at rest (L/min). To assess physical performance, patients were asked how many stairway steps they can cover without having to rest. More liters of supplemental oxygen and a lower number of steps were considered as being indicative of a higher degree of severity of the somatic disease (18).

### Statistics

In a study of Hoffman et al. (2), the English version of the PQLS demonstrated a three-factor structure using exploratory factor analysis (EFA). This structure was tested in the current sample with a CFA. Furthermore, we fit an additional one-factor model (referring to a general QoL dimension) (19) and evaluated the fit of both models taking the fit indices described below into account. A maximum of 10% missing data (two missing items) per person was allowed in the PQLS. The reported CFA were also conducted with *n* = 140 participants for both the one- and the three-factor model. This was possible thanks to the “pairwise-deletion” option in the lavaan R-package which allows for all available information to be used in the CFA (20, 21).

Parameters of the CFA models were estimated with a WLSMV (weighted least squares with mean and variance adjusted) estimator that uses diagonally weighted least squares (DWLS) as well as mean and variance adjusted test statistics as the PQLS is skewed and measured on an ordinal scale (22). The analyses were performed in R 3.4.4 (21) using the lavaan package (20). Model fit was assessed by means of multiple criteria: chi-square test statistic for absolute fit, comparative fit index (CFI) for fit relative to a null model, complemented with the (standardized) root mean square residual (SRMR) and the root mean square error of approximation (RMSEA) for overall fit. The criteria for good model fit are defined according to Hu and Bentler (23) as CFI > 0.95 (0.90 is acceptable), SRMR < 0.06 (0.09 is acceptable), and RMSEA < 0.06 (0.09 is acceptable).

Internal consistency of the PQLS was assessed by calculating the Cronbach’s α coefficient for the total score and for each of the subscales. Relationships between the subscales were determined using two-tailed Spearman’s rank-order correlations.

Convergent validity was assessed by examining Pearson correlation coefficients between the PQLS total score and the scores on the generic HRQoL instruments (SF-8, 10-point scale), measures of depression and anxiety (PHQ-9, GAD-7), and functional measures of lung disease severity (oxygen use and steps). Correlation coefficients ≥0.1 were interpreted as a low correlation, coefficients ≥0.3 as a moderate correlation, and coefficients ≥0.5 as a strong correlation.

Sensitivity to change after transplantation was evaluated using dependent sample *t*-tests with time as within-person variable and PQLS as dependent variable. To estimate the effect size of the differences, Cohen’s *d* was calculated.

Finally, we examined the association between age, sex, civil status, diagnostic groups—as described above—and the PQLS total and subscale scores. Mann–Whitney U-tests and Kruskal–Wallis tests were used for comparison of continuous data and chi-square analyses for categorical data.

Statistical analyses were performed using IBM Statistical Software Package of Social Science Statistics version 24 and R 3.4.4, as appropriate. For all analyses, *p* < 0.01 was considered statistically significant.

## Results

### Description of the Sample

Participants’ characteristics are summarized in **Table 1**. One hundred fifty patients participated in the study. However, 10 of the 150 participants had more than 10% missing data (more than two missing items) in the PQLS and were excluded from further analyses resulting in a final sample size of 140 lung transplant candidates with valid PQLS data. The sample consisted of 63 women (45%) and 77 men (55%). The median age was 53 years [Interquartile range (IQR) 13]. Twenty-eight point six percent of the participants reported an educational level of 12 or more years of school attendance. About 80.7% stated to be married or to be living in a partnership. In the sample, the most common lung disease category was restrictive (30%), followed by cystic fibrosis (27.9%), obstructive (21.4%), and others (20.7%).

**Table 1:** Characteristics of study participants.

	Total	Obstructive	CF	Restrictive	Other
*N* (%)	140 (100%)	30 (21.4%)	39 (27.9%)	42 (30.0%)	29 (20.7%)
Age, years, mean (SD) median (IQR)	49.9 (12.3) 53.0 (14)	56.9 (4.8) 57.5 (7)	39.6 (13.8) 42.0 (26)	57.4 (5.6) 58.0 (8)	45.5 (11.1) 47.0 (16)
Sex, women % (*n*)	45.0 (63)	33.3 (10)	48.7 (19)	33.3 (14)	69.0 (20)
Educational level ≥12 years % (*n*)	28.6 (40)	16.7 (5)	41.0 (16)	26.2 (11)	27.6 (8)
Living in a partnership % (*n*)	80.7 (113)	76.7 (23)	66.7 (26)	97.6 (41)	79.3 (23)
Number of stairway steps mean (SD) median (IQR)	8.3 (6.4) 7.0 (6.4)	6.4 (6.4) 5.0 (6.1)	10.7 (7.6) 10.0 (10.0)	7.8 (4.7) 7.0 (5.4)	7.7 (6.0) 6.0 (5.4)
Oxygen use at rest (l/min) mean (SD) median (IQR)	2.2 (1.6) 2.0 (2.0)	2.3 (1.7) 2.0 (0.5)	1.7 (1.2) 2.0 (2.0)	2.7 (1.8) 2.5 (2.3)	1.9 (1.5) 2.0 (2.5)
PHQ-9 mean (SD) median (IQR)	7.1 (4.2) 6.0 (7.0)	7.2 (4.5) 6.9 (7.2)	7.6 (4.2) 8.0 (6.1)	7.3 (4.4) 6.0 (7.0)	5.9 (3.8) 5.0 (5.5)
GAD-7 mean (SD) median (IQR)	3.7 (2.9) 3.0 (5.0)	3.8 (3.2) 3.0 (5.0)	3.7 (2.8) 3.0 (5.0)	3.8 (3.1) 3.0 (5.5)	3.6 (2.6) 3.0 (4.0)
PQLS, Total score mean (SD) median (IQR)	85.3 (14.1) 87.3 (19.8)	90.4 (11.3) 91.5 (17.0)	82.1 (14.4) 84.0 (21.0)	85.7 (14.5) 87.3 (20.5)	83.9 (15.0) 84.0 (17.9)
PQLS, Task interference mean (SD) median (IQR)	32.5 (5.4) 33.1 (8.1)	34.2 (4.8) 36.0 (8.3)	31.5 (5.9) 33.0 (8.0)	32.9 (4.9) 34.3 (7.0)	31.6 (6.0) 32.5 (7.5)
PQLS, Psychological mean (SD) median (IQR)	18.6 (5.5) 18.0 (8.0)	20.1 (5.4) 20.0 (8.0)	18.3 (4.6) 17.5 (6.0)	18.6 (6.6) 18.0 (11.0)	17.4 (5.2) 17.0 (7.5)
PQLS, Physical mean (SD) median (IQR)	17.3 (4.1) 19.0 (4.0)	17.4 (4.8) 19.5 (2.5)	16.3 (4.1) 17.0 (6.7)	17.7 (3.2) 19.0 (4.0)	17.7 (4.2) 20.0 (1.5)
SF-8, PCS mean (SD) median (IQR)	32.1 (7.4) 31.8 (9.9)	29.7 (5.7) 29.5 (7.5)	33.0 (9.0) 31.7 (13.5)	32.9 (6.1) 32.1 (9.4)	32.1 (8.0) 33.0 (11.1)
SF-8, MCS mean (SD) median (IQR)	46.5 (10.7) 46.8 (14.2)	44.8 (11.3) 45.0 (16.3)	44.1 (10.4) 43.8 (15.4)	49.0 (11.0) 49.4 (17.2)	48.3 (9.5) 48.7 (14.0)
Subjective quality of life (scale 0 to 10) mean (SD) median (IQR)	3.7 (1.9) 3.0 (3.0)	2.7 (1.2) 2.5 (1.0)	3.7 (2.0) 3.0 (3.0)	4.2 (1.9) 4.0 (2.0)	4.0 (1.9) 4.0 (3.0)

PQLS, Pulmonary-Specific Quality-of-Life Scale; SF-8, Short Form 8 Health Survey; PCS, Physical Component Scale; MCS, Mental Component Scale; GAD-7, Generalized Anxiety Scale; PHQ-9, Patient Health Questionnaire-Depression Scale; CF, cystic fibrosis; IQR, interquartile range.

### Factor Structure

The result of the CFA with three factors and a single factor can be found in **Table 2**. For the three-factor model RSMEA and CFI denoted a good fit, but the SRMR reached a value above the acceptable cutoff; however, the SRMR reaches higher values in studies with small sample sizes and lower degrees of freedom (24). The other measures indicated a good fit; thus, we concluded that the three-factor model obtained an acceptable fit. For the single-factor model, the CFI showed an acceptable fit, while SRMR and RSMEA reached values above the acceptable cutoff. Therefore, the single-factor model did not show an acceptable fit.

**Table 2:** Fit indices for the confirmatory factor analysis of the PQLS (weighted scores).

	Chi²	df	CFI	SRMR	RMSEA
PQLS single-factor	455.17 ( *p* < 0.01)	275	0.846	0.149	0.083
PQLS three-factor	203.875 ( *p* < 0.01)	149	0.967	0.113	0.051

CFI, comparative fit index; SRMR, (standardized) root mean square residual; RMSEA, root mean square error of approximation; df, degrees of freedom.

Factor loadings can be found in **Table 3**. Item 23 (“I have been embarrassed by coughing in public.”) obtained a rather low item loading (0.16). We decided to keep this item in the final solution to be able to compare our results with previous findings. The factor correlations were 0.40 between “psychological” and “task interference,” 0.61 between “task interference” and “physical,” and 0.32 between “psychological” and “physical.”

**Table 3:** Factor loadings of the three-factor structure of the PQLS.

Item	Factor “Task interference”	Item	Factor “Psychological”	Item	Factor “Physical function”
9	0.78	5	0.70	1 (reverse scored)	0.74
11 (reverse scored)	0.74	6	0.62	2 (reverse scored)	0.96
12 (reverse scored)	0.71	7	0.64	3 (reverse scored)	0.95
13	0.73	20	0.48	4 (reverse scored)	0.81
15	0.34	23	0.16		
16	0.67	24	0.63		
21	0.60	25	0.77		
22	0.70				

### Reliability of the Pulmonary-Specific Quality-of-Life Scale

For all scales, the internal consistency coefficients turned out acceptable ranging from 0.70 to 0.85 (**Table 4**). Cronbach’s α for the PQLS total score was 0.82, indicating a good reliability. Spearman rank correlations between the subscales can be found in **Table 5**.

**Table 4:** Internal consistencies of the PQLS.

	Cronbach’s α
PQLS total	0.82
Subscales Task interference Psychological Physical	0.70 0.73 0.85

**Table 5:** Spearman rank correlation between PQLS subscales.

	Psychological	Physical
Task interference	0.24**	0.49**
Psychological		0.23**
Physical		–

**p < 0.01.

### Validity of the Pulmonary-Specific Quality-of-Life Scale

#### Convergent Validity of the Pulmonary-Specific Quality-of-Life Scale With Other Measures of Quality of Life, Depression, and Anxiety

In **Table 6**, the relationships between the PQLS total and subscale scores, levels of HRQoL using generic measures, and levels of depression and anxiety are reported.

**Table 6:** Relationship between the PQLS total and subscale scores and physical functioning, generic measures of health-related quality of life, levels of depression and anxiety.

	PQLS total	Task interference	Psychological	Physical
Number of stairway steps	−0.40**	−0.42**	−0.11	−0.26**
Oxygen use at rest (l/min)	0.19	0.25**	−0.05	0.23**
Subjective QoL (scale 0 to 10)	−0.52**	−0.45**	−0.31**	−0.31**
GAD-7	0.42**	0.08	0.65**	0.08
PHQ-9	0.41**	0.21	0.57**	0.00
SF-8 PCS MCS	−0.57** −0.42**	−0.61** −0.16	−0.22 −0.61**	−0.32** −0.05

QoL, quality of life. **p < 0.01.

The PQLS total score correlated significantly and negatively with other HRQoL measures as well as positively with symptoms of depression and anxiety. All three PQLS subscales correlated significantly with the 10-point self-reported QoL scale, with *r* ranging between −0.31 and −0.45 (*p* < 0.001) (negative correlation due to the opposed alignment of the HRQoL measures in comparison to the PQLS). Low values on the PCS of the SF-8, indicating poor physical functioning, were significantly and strongly correlated with high values in the subscale “task interference,” representing significant impairment (*r* = −0.61, *p* < 0.001). The PCS was also significantly correlated with the PQLS subscale “physical” (*r* = −0.32, *p* < 0.001), although this relationship was quite modest. Additionally, we found a significant and strong relationship between the MCS and the PQLS subscale “psychological” (*r* = −0.62, *p* < 0.001).

The subscale “psychological” correlated significantly and positively with symptoms of anxiety (*r* = 0.65) and depression (*r* = 0.57) (both *p* < 0.001).

#### Convergent Validity of the Pulmonary-Specific Quality-of-Life Scale With Measures of Disease Severity and Functional Capacity

The PQLS total score was significantly associated with measures of disease severity (**Table 6**), with higher (worse) PQLS scores associated with fewer stairway steps without rest (*r* = −0.4). Specifically the PQLS subscales “task interference” and “physical” correlated significantly with the number of stairway steps (*r* = −0.42, *p* < 0.001 and *r* = −0.26, *p* = 0.002) and supplemental oxygen use (*r* = 0.25, *p* = 0.004 and *r* = 0.23, *p* = 0.006).

### Sensitivity to Change After Transplantation

Of the 140 participants who provided complete baseline data, 43 have already completed the assessment 1 year after transplantation. The characteristics of the participants can be found in **Table 7**. The sample consisted of 17 women (39.5%) and 26 men. The median age was 52 years (IQR 16) at baseline. Overall, 20.9% had been suffering from an obstructive lung disease, 34.9% from cystic fibrosis, 25.6% from a restrictive disease, and 18.6% from a different lung disease.

**Table 7:** Participants’ characteristics after transplantation.

	Total
*N* (%)	43 (100%)
Medical condition, *n* (%) obstructive CF restrictive other	9 (20.9%) 15 (34.9%) 11 (25.6%) 8 (18.6%)
Age, years mean (SD) median (IQR)	47.8 (13.5) 52 (16)
Sex, women, *n* (%)	17 (39.5)
Educational level ≥12 years, *n* (%)	9 (20.9)

IQR, interquartile range.

PQLS total scores declined from a mean value of 84.2 (SD 14.6) before transplantation to a mean value of 45.6 (SD 13.9) (*t*(42) = 13.105, *p* < 0.001) after transplantation (**Table 8**). This corresponds to a large effect (*d* = 2.702). A significant and large improvement could also be found for each of the three subscales: “task interference” (*t*(39) = 10.204, *p* < 0.001, *d* = 2.193), “psychological” (*t*(42) = 7.882, *p* < 0.001, *d* = 1.545), and “physical” (*t*(42) = 10.366, *p* = 0.001, *d* = 2.284).

**Table 8:** Comparison before and after transplantation.

	Baseline [Mean (SD)]	Follow-up [Mean (SD)]	Statistics	Effect size
PQLS total	84.2 (14.6)	45.6 (13.9)	*t* = 13.105, *p* < 0.001, 95% CI: [32.64, 44.53]	*d* = 2.702
Subscales Task interference Psychological Physical	32.2 (6.3) 18.5 (5.6) 17.0 (4.4)	17.6 (6.9) 10.6 (4.6) 6.9 (4.5)	*t* = 10.204, *p* < 0.001, 95% CI: [11.70, 17.49] *t* = 7.882, *p* < 0.001, 95% CI: [5.85, 9.87] *t* = 10.366, *p* < 0.001, 95% CI: [8.18, 12.14]	*d* = 2.193 *d* = 1.545 *d* = 2.284
SF-8 PCS MCS	30.8 (8.5) 46.7 (10.4)	50.6 (8.7) 54.5 (6.7)	*t* = −11.322, *p* < 0.001, 95% CI: [−23.35, −16.27] *t* = −3.840, *p* < 0.001 95% CI: [−11.91, −3.69]	*d* = 2.305 *d* = 0.899

d, effect size for dependent t-tests (25).

Additionally, a significant change could be found for the PCS [30.8 (8.5) vs. 50.6 (8.7), *t*(39) = −11.322, *p* < 0.001, *d* = 2.305] and for the MCS of the SF-8 [*t*(39) = −3.840, *p* < 0.001, *d* = 0.899].

### Association With Age, Sex, Partnership Status, and Diagnostic Group

There were no significant differences in the PQLS total and three subscale scores between female and male participants. Also, no difference in PQLS total and subscale scores were detected between participants with 12 or more years of school attendance and those with less than 12 years.

Higher age was significantly but weakly related to a higher PQLS total score (*r* = 0.230, *p* = 0.006), representing lower HRQoL. There was no statistically significant correlation between age and the three subscales.

There was no statistically significant difference regarding the PQLS total score or its subscales in participants living in a partnership (*n* = 113) compared to those living without a partner.

The four diagnostic groups did not differ significantly with regard to the PQLS total score and the PQLS subscale scores (**Table 1**). Additionally, there were no differences between the diagnostic groups regarding HRQoL measured with the SF-8, symptoms of depression and anxiety, supplemental oxygen at rest, and educational level.

## Discussion

The main finding of our study was that the original three-factor model could be replicated in the German version of the PQLS in patients with end-stage lung disease awaiting lung transplantation. The PQLS total score and the three PQLS subscales “physical,” “psychological,” and “task interference” showed acceptable internal consistencies. Overall, the mean values of the PQLS and its subscales were comparable to the study of Hoffman et al. (2).

Convergent validity was established through correlations between the PQLS total and the three subscale scores with established measures of generic HRQoL. The PQLS total score correlated significantly with the measures of generic HRQoL in the expected direction. The strongest association was found between the PQLS subscale “psychological” and the MCS of the SF-8 highlighting that this subscale assesses mental aspects of HRQoL. An equally strong link was found between the PQLS subscale “task interference” and the PCS of the SF-8, indicating that this subscale measures physical impairment.

Regarding measures of depression and anxiety, we found significant associations between higher values of anxiety and depression and the PQLS total score as well as the subscale “psychological.” This finding supports the criterion validity of the PQLS given that lower HRQoL in lung disease patients is known to be associated with higher levels of anxiety and depression (5).

Taking measures of disease severity into account, higher lung disease severity, as indicated by a higher oxygen use, and less covered stairway steps were significantly and moderately associated with higher (more pathological) scores in the subscales “task interference” and “physical.” These findings are in accordance with the results of Hoffman et al. (2) and show that disease severity correlates primarily with measures indicating physical impairment, and not with psychological and mental aspects.

Regarding sociodemographic parameters, there were no significant associations between sex, years of school attendance, and pulmonary-specific HRQoL. However, older age was associated with lower HRQoL. No statistically significant association between partnership status and HRQoL was found. No significant differences in PQLS scores were found between the diagnostic groups.

After transplantation, there was a significant improvement in the total and all three subscale scores of the PQLS, with large effect sizes for the scales. These findings are in accordance with the results of Hoffman et al. (2).

The strengths of our study are the use of a CFA to test the factor structure of the PQLS and the comparison of the HRQoL before and after lung transplantation.

However, there are some limitations. The number of patients who were assessed before and after transplantation made up only about one third of the participants at baseline. The 43 patients who underwent a successful lung transplantation reported a large reduction of nearly 50% of their PQLS scores corresponding with a large effect size. However, our study did not allow determining the minimally clinically important difference (MCID) for the PQLS (26), the smallest change in PQLS scores, which can be considered to be clinically important for the patient, due to the small sample size and the considerable difference before and after transplantation found in all patients. Also, supplemental oxygen use and stairway steps may not give a complete picture of disease severity, especially as both parameters are patient-reported data.

In conclusion, the results suggest that the German version of the PQLS has good reliability, validity, and sensitivity to change after transplantation. It constitutes a suitable and easy to apply instrument to assess HRQoL in patients with end-stage lung disease awaiting lung transplantation. These medical conditions come along with unique physical and mental burdens which can be measured using the PQLS. To our knowledge there are no instruments available fulfilling this purpose so far. Future studies with a larger sample size before and after transplantation will be helpful to confirm the results of our study. Additionally, the PQLS should be used not only 1 year after transplantation but also after a longer follow-up period.

## Ethics Statement

The study was approved by the Ethics committee of Hannover Medical School (no 3120-2016). All participants signed an informed consent in accordance with the Declaration of Helsinki.

## Author Contributions

MZ designed the study. ÖA was mainly responsible for data acquisition. MZ and MN analyzed the data, and MN wrote the first draft. LC, DS, and JB performed the CFA and supported the data analysis. All authors contributed significantly to the interpretation of the data and the final version of the manuscript. All authors gave final approval of the version to be published.

## Funding

The study was supported by a grant from Biomedical Research in Endstage and Obstructive Lung Disease Hannover (BREATH) within the German Center for Lung Research (82DZL002A1).

## Conflict of Interest Statement

All authors declare that the research was conducted in the absence of any commercial or financial relationship that could be construed as a potential conflict of interest.
